# Progressive Encephalomyelitis with Rigidity and Myoclonus Associated With Anti-GlyR Antibodies and Hodgkin’s Lymphoma: A Case Report

**DOI:** 10.3389/fneur.2017.00401

**Published:** 2017-08-10

**Authors:** Linda Borellini, Silvia Lanfranconi, Sara Bonato, Ilaria Trezzi, Giulia Franco, Lorella Torretta, Nereo Bresolin, Alessio Barnaba Di Fonzo

**Affiliations:** ^1^Neurology Department, IRCCS Fondazione Cà Granda Ospedale Maggiore Policlinico, Milan, Italy; ^2^Dino Ferrari Center, University of Milan, Milan, Italy; ^3^Aferesi Terapeutica, Centro Trasfusionale, Fondazione IRCCS Cà Granda Ospedale Maggiore Policlinico, Milan, Italy

**Keywords:** progressive encephalomyelitis with rigidity and myoclonus, glycine receptor antibodies, paraneoplastic syndromes, stiff person syndrome, Hodgkin’s lymphoma

## Abstract

**Introduction:**

A 60-year-old man presented with a 6-month history of low back pain and progressive rigidity of the trunk and lower limbs, followed by pruritus, dysphonia, hyperhydrosis, and urinary retention. Brain and spinal imaging were normal. EMG showed involuntary motor unit hyperactivity. Onconeural, antiglutamic acid decarboxylase (anti-GAD), voltage-gated potassium channel, and dipeptidyl peptidase-like protein 6 (DPPX) autoantibodies were negative. CSF was negative. Symptoms were partially responsive to baclofen, gabapentin, and clonazepam, but he eventually developed severe dysphagia. Antiglycine receptor (anti-GlyR) antibodies turned out positive on both serum and CSF. A plasmapheresis cycle was completed with good clinical response. A PET scan highlighted an isolated metabolically active axillary lymphnode that turned out to be a classic type Hodgkin lymphoma (HL), in the absence of bone marrow infiltration nor B symptoms. Polychemotherapy with ABVD protocol was completed with good clinical response and at 1-year follow-up the neurological examination is normal.

**Background:**

Progressive encephalomyelitis with rigidity and myoclonus (PERM) is a rare and severe neurological syndrome characterized by muscular rigidity and spasms as well as brain stem and autonomic dysfunction. It can be associated with anti-GAD, GlyR, and DPPX antibodies. All of these autoantibodies may be variably associated with malignant tumors and their response to immunotherapy, as well as to tumor removal, is not easily predictable.

**Conclusion:**

Progressive encephalomyelitis with rigidity and myoclonus has already been described in association with HL, but this is the first case report of a HL manifesting as anti-GlyR antibodies related PERM. Our report highlights the importance of malignancy screening in autoimmune syndromes of suspected paraneoplastic origin.

## Introduction

A 60-year-old previously healthy man was referred to our hospital for subacute onset of severe and progressive gait disturbance associated with painful muscular spasms of the trunk and lower limbs. Symptoms appeared 5 months before with lumbar and left leg pain, followed by progressive gait difficulty especially in climbing stairs. At that time a first neurological examination was unremarkable except for positive Lasègue sign on the right side; 75 mg of oral prednisone was prescribed, with a partial relief of symptoms. Three months after symptoms, onset spontaneous and stimulus sensitive muscular spasms appeared, and an EMG confirmed involuntary continuous motor unit activity of all examined muscles. At that time a neurological examination disclosed bilateral achilleus clonus associated with clubfoot and mallet fingers. Blood creatine kinases were slightly increased (up to 518 U/L), while autoantibodies [ANA, ENA, anti Hu-Yo-Ri-Amphiphysin-CV2-Ma2/Ta, and antiglutamic acid decarboxylase (anti-GAD)] were negative. Cerebrospinal fluid examination was normal. In order to rule out paraneoplastic syndromes, a total body CT scan was performed, showing two small axillary lymph nodes (defined as reactive) and a diffuse swelling and edema of left psoas, iliacus, pectineus, obturator externus, and quadratus lumborum, which was attributed to focal myositis. He was then treated with intravenous methylprednisolone followed by 1 mg/kg of oral prednisone. Despite treatment, he became wheelchair bound and was sent to rehabilitation, where he developed progressive dysphonia and diffuse pruritus on steroid tapering. For this reason he was admitted to our hospital.

Neurological examination showed marked dysphonia with rhynolalia and slight bilateral facial weakness. Ocular movements were unaffected. Marked truncal and proximal lower limb rigidity, with incapacity to sit and stand unassisted, was noticed. Diffuse hyperreflexia with knee and ankle clonus was elicited. Cutaneous plantar reflex was flexor. He also had diffuse hyperhydrosis, constipation, and urinary retention. A therapy with oxibutinine and tamsulosine was prescribed, but he still required frequent autocatheterization. No muscular weakness, cerebellar signs, nor sensory defects were detected. However, he complained of severe dysesthesias and diffuse pruritus. After admission, he also developed severe dysphagia, requiring a feeding tube.

**Figure 1:**
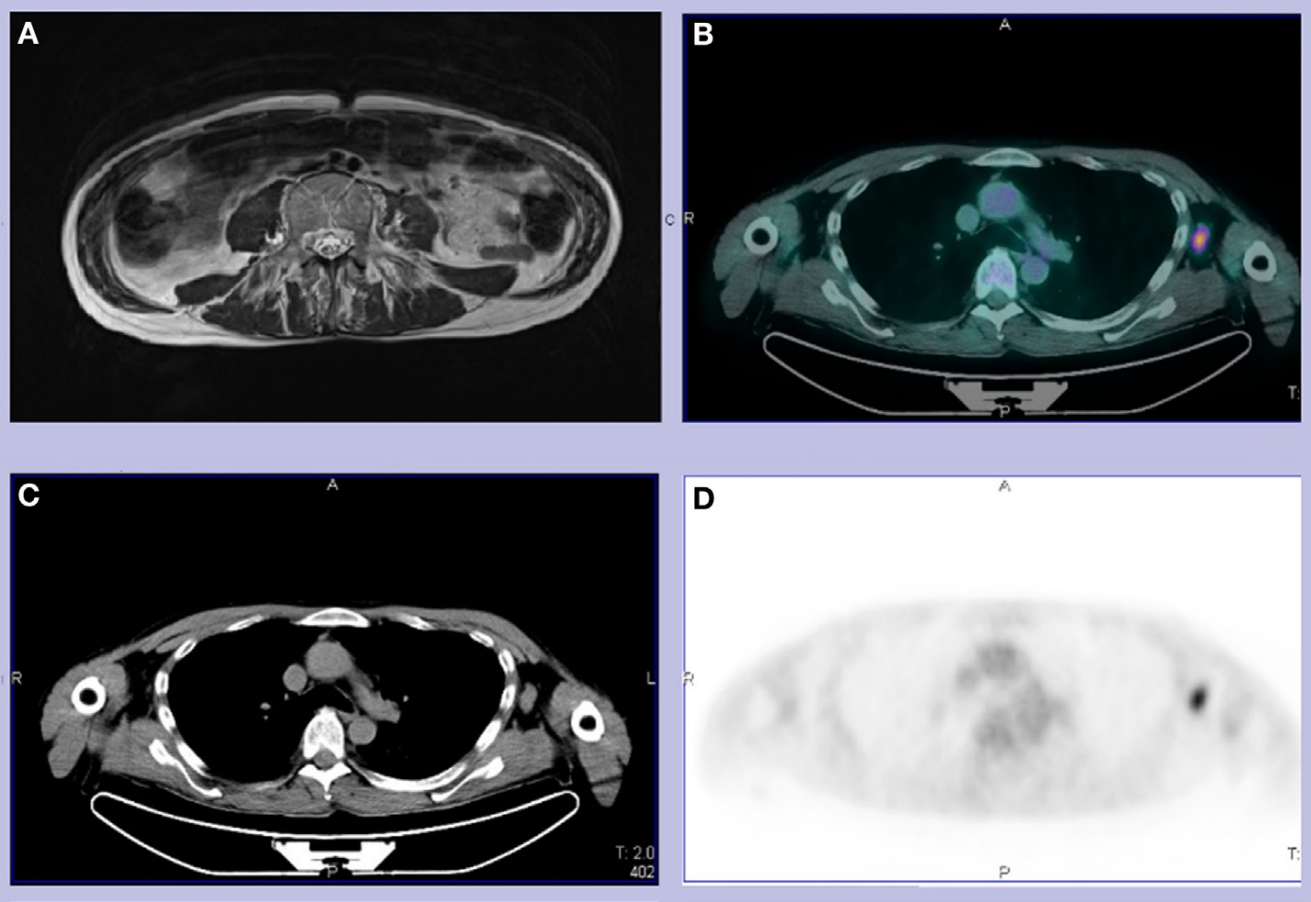
**(A)** T2 abdominal MRI revealing left psoas and bilateral paravertebral muscles edema and fatty substitution. **(B–D)** Total body CT-PET showing a hypermetabolic left axillary lymph node. We did not use copyrighted material from other sources (including the web).

EMG was unremarkable, also in the paravertebral muscles. There was an increased cortical latency at both motor and sensory evoked potentials at the four limbs but brain and spinal MRI showed no abnormality except from the muscular swelling that had been mentioned at the first CT (Figure 1A). EEG was normal and there were no cognitive or psychiatric symptoms. A PET scan highlighted one of the two previously reported axillary lymph nodes as being enlarged and metabolically active (Figures 1B–D).

Symptoms markedly improved with clonazepam (up to 2.5 mg/day) and gabapentin (up to 1.200 mg/day). Steroid dosage increase up to 50 mg lead to a partial relief of sensory symptoms.

Blood tests were normal, including previously tested autoantibodies, voltage-gated potassium channel complex, and dipeptidyl peptidase-like protein 6 (DPPX) antibodies. GAD antibodies were still negative, as well as CSF examination, including protein level, IgG, and OGB. Finally, anti–GlyR (antiglycine receptor) antibodies were found to be positive on both serum and CSF samples, respectively. Meanwhile the patient started a plasmapheresis treatment, five cycles, 3,000 mL/cycle.

The metabolically active anxillary lymph node was removed and its histological examination was consistent with a classic type Hodgkin’s lymphoma. Of note, there was no bone marrow infiltration, no elevation in β2-microglobulin, nor lactate dehydrogenase in blood. Partial clinical improvement was observed 10 days after plasmapheresis. The patient was then able to stand unassisted and walk with minimal help. Dysphagia completely resolved few weeks after. He eventually was transferred at the Oncoematology Division to undergo polychemotherapy with ABVD protocol (doxorubicin, bleomycin, vinblastine, and dacarbazine). Over a 12-month follow-up period, a constant clinical improvement was observed despite tapering of immunosuppressive and symptomatic treatment. The patient was able to walk unassisted, to carry out usual activities including a full time job. He was still symptomatic for inconstant self-resolving stiffness in the morning (12 months mRS score was 1). A control PET scan was negative. The abdominal and pelvic muscles had a normal MRI appearance.

Written informed consent was obtained from the participant for the publication of this case report.

## Background

Progressive encephalomyelitis with rigidity and myoclonus (PERM) was first described 40 years ago by Whiteley et al. (1). It was also called stiff person plus syndrome, sharing some of its core clinical features and being sometimes associated with GAD autoimmunity. In 2008, anti-GlyR antibodies were detected in a typical PERM patient with hyperekplexia, rigidity, and brain stem signs (2), and in the following years, more cases were reported (3–8). An association with tumor has been reported in about 20% of cases (9) Recently, a novel antibody, namely anti-DPPX was reported to be associated with PERM with frequent gastrointestinal involvement (10–12). Another case report associates PERM to amphiphysin antibodies (13).

All of these autoantibodies have been related in variable frequency to malignant tumors and their response to immunotherapy, as well as tumor removal, is also not easily predictable (9–14).

## Discussion

To our knowledge, this is the first case report of a Hodgkin’s lymphoma manifesting as glycine-receptor antibodies related PERM. In their prospective analysis of 45 GlyR antibodies positive patients, Carvajal-Gonzàlez et al. reported a case of PERM/myastenia gravis plus syndrome in a 51-year-old male with a past history of successfully treated Hodgkin’s lymphoma (14). Schmidt et al. describe a patient presenting with generalized pruritus, sleep disturbance and paroxismal fear, followed by rapidly progressive gait ataxia, generalized myoclonic jerks, severe dysautonomia, and respiratory failure who eventually developed a Hodgkin’s lymphoma (15). There was no screening for antibodies against GlyR; however, the presence of status epilepticus and psychiatric features might also suggest an NMDAR encephalitis. In this regard, an overlap of the syndromes associated with both GlyR and NMDAR antibodies was described (5). The absence of seizures, psychiatric symptoms, and signs of hippocampal involvement in our patient did non-prompt us to investigate NMDAR autoimmunity. McKeon et al. also report a case of stiff person syndrome with GlyR antibodies in a young man who 12 years later was diagnosed with Hodgkin’s lymphoma. However, he never developed signs of brain stem involvement and the timing of the malignancy diagnosis makes this association questionable (16). Our patient did not complain systemic symptoms of malignancy. However, hyperhydrosis and itching, that are common in non-paraneoplastic PERM (14), could be interpreted as B symptoms as well. At CT scan, the lymphoma was overlooked, being described as a probably reactive lymph node. This highlights the importance of performing a more sensitive PET scan in patients with a strong suspect of a paraneoplastic syndrome.

Imaging showed atypical features in the abdominal and pelvic muscles. Such findings have not been described before and were partly misleading for diagnosis and treatment, since they suggested a focal myositis. The muscular edema resolving soon after symptomatic therapy rather than with immunotherapy suggests a possible association to hypercontraction rather than an inflammatory process. Interestingly, we noticed a similar CT and MRI muscle images in a young woman with anti-GAD-related stiff person syndrome with myopathic discharges at EMG, which eventually evolved into fibrous substitution.

In summary, we presented a case of PERM with GlyR antibodies as first manifestation of Hodgkin’s lymphoma. A correct interpretation of symptoms and imaging investigations is of primary importance to rule out an underlying occult malignancy and speed up the appropriate treatment.

## Ethics Statement

The study was exempt from this requirement. Non-applicable. The current study does not involve vulnerable populations.

## Author Contributions

LB and SL: clinical assessment, acquisition of data, manuscript drafting. SB, IT, GF, and LT: clinical assessment, acquisition of data. NB: clinical assessment, critical revision of manuscript. AD: critical revision of manuscript.

## Conflict of Interest Statement

The authors declare that the research was conducted in the absence of any commercial or financial relationships that could be construed as a potential conflict of interest.
